# Mechanical Characterization and Thermodynamic Analysis of Laser-Polished Landscape Design Products Using 3D Printing

**DOI:** 10.3390/ma14102601

**Published:** 2021-05-17

**Authors:** Yue Ba, Yu Wen, Shibin Wu

**Affiliations:** 1 College of Arts & Design, Yanshan University, Qinhuangdao 066004, China; Bayueyanshan@163.com; 2 Three Gorges New Energy Kangbao Power Generation Co., Ltd., Zhangjiakou 076650, China; wu_shibin@163.com

**Keywords:** 3D printing, landscape design, material characterization, mechanical property, laser polishing

## Abstract

Recent innovations in 3D printing technologies and processes have influenced how landscape products are designed, built, and developed. In landscape architecture, reduced-size models are 3D-printed to replicate full-size structures. However, high surface roughness usually occurs on the surfaces of such 3D-printed components, which requires additional post-treatment. In this work, we develop a new type of landscape design structure based on the fused deposition modeling (FDM) technique and present a laser polishing method for FDM-fabricated polylactic acid (PLA) mechanical components, whereby the surface roughness of the laser-polished surfaces is reduced from over Ra 15 µm to less than 0.25 µm. The detailed results of thermodynamics and microstructure evolution are further analyzed during laser polishing. The stability and accuracy of the results are evaluated based on the standard deviation. Additionally, the superior tensile and flexural properties are examined in the laser-polished layer, in which the ultimate tensile strength (UTS) is increased by up to 46.6% and the flexural strength is increased by up to 74.5% compared with the as-fabricated components. Finally, a real polished landscape model is simulated and optimized using a series of scales.

## 1. Introduction

Landscape design is not only a symbol of urbanization, but also an embodiment of urban culture and charm. New landscape architecture approaches have triggered the emergence of a large number of public development spaces [1,2]. Traditional landscape production methods are increasingly unable to meet the efficiency requirements and diverse characteristics by modern society [3]. In order to re-use these spaces to enhance the cultural aspects of urbanization, we present a landscape design concept that should transform the current state of this field, improving efficiency and reducing environmental pollution risk in landscape architecture [4,5].

Globally, 3D printing (additive manufacturing) is one of the new information manufacturing technologies that is promoting automation of traditional landscape sculptures, which is impacting manufacturing ecosystems, as the supply chain in the process of landscape construction can be minimized [6,7,8]. The freedom building approach increases fabrication flexibility, offering a wide range of advantages, such as net shape fabrication, increased product customization, and high production efficiency [9]. Therefore, this technology has been widely used in aerospace, biomedical industries, landscape construction, and marine and offshore applications [6]. Although there has been significant research on 3D printing for landscape construction in the last three decades, work on the design of polymeric materials for landscape applications is still limited [10,11].

**Figure 1:**
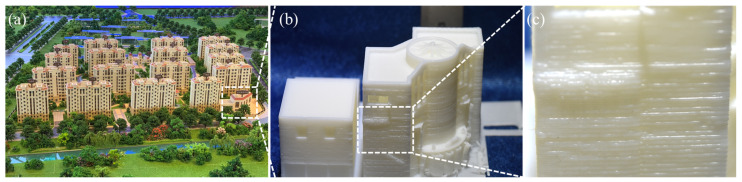
Landscape design photographs of a 3D-printed PLA model set of buildings in Qinhuangdao, China: (**a**) model set of landscape design and buildings; (**b**,**c**) locally enlarged graphs.

As one of the most popular 3D printing technologies, fused deposition modeling (FDM) is mainly used to produce thermoplastic materials, such as acrylonitrile-butadiene-styrene (ABS), high-impact polystyrene (HIPS), and polylactic acid (PLA, [12,13,14]. Karimi et al. produced an environmentally friendly stone composite based on ABS and recycled natural granite stone particles, which could be considered as a potential material for 3D printing [15]. As such, we adopted a research strategy, in which we sought out exemplary organizations within real estate in order to understand the changes that 3D printing has brought to their operations. A model set of buildings in Qinhuangdao, China, was printed using FDM and PLA materials, which can be seen in Figure 1a,b. Unfortunately, high surface roughness and porosity usually occur in such freeform-fabricated components due to the staircase effect, non-uniform temperatures, and thermal shrinkage or expansion [16,17,18], which can be seen in Figure 1c. Therefore, such products usually requires additional post-treatment or parameter optimization to meet landscape design requirements.

Although there have been many studies on parameter optimization, such as the study by Khosravani et al., which presented the influence of two printing parameters on the strength of 3D-printed components [19], solutions for the staircase effect and the occurrence of non-uniform temperature in the FDM formation process are still required [20,21]. Compared with traditional surface post-treatment technologies, such as mechanical polishing and chemical mechanical polishing, laser polishing, as an emerging method, can improve the surface integrity of metal and non-metal components via re-melting the thermal input of the laser irradiation and rapid smooth solidification [22,23]. In recent years, laser polishing has been successfully employed to improve the surface finish of components fabricated using 3D printing due to its non-contact mode of operation, high efficiency, precision, and lack of mechanical damage [22,24].

In the literature, laser polishing has been applied for various metal 3D printing components, e.g., CoCr alloy [25,26,27], nickel-based superalloys [28], titanium alloy [29,30,31], and stainless steel [32,33], in order to reduce surface roughness and enhance surface properties. For polymer 3D printing components, Taufik et al. [34] first adopted a pulsed CO_2_ laser source to polish ABS components fabricated via FDM. The changes in roughness parameters were analyzed before and after the laser polishing process. Chai et al. [35] used a CO_2_ laser to polish PLA surfaces, whereby the Ra value of the surface roughness decreased by 68%. Chen et al. [36] investigated the surface quality and mechanical properties of laser-polished, FDM-fabricated PLA specimens, showing that the surface roughness could be reduced by 90.4%. Additionally, Chen et al. [21] further studied the laser polishing process for copper fiber–polylactide acid (Cu/PLA) composite parts manufactured using FDM, showing that the storage modulus, loss modulus, and glass transition temperature of Cu/PLA composites were significantly improved. Lambiase et al. [20] used a 30 W CO_2_ laser source to polish the surfaces of components made of PLA and obtained via material extrusion (ME), for which the Ra value of the surface roughness was reduced from 12 µm to 0.3 µm. Note that previous studies have shown that the surface roughness of FDM-fabricated PLA specimens can be decreased from over 12 μm to 0.3 μm by using a continuous-wave fiber laser or a pulsed CO_2_ laser source. It should be noted that there have been very few studies involving thermodynamic analysis of and rapid solidification during the laser polishing polymer process, which is essential for surface finishing and the resulting properties. These surface properties are directly related to the aesthetic quality and service life of 3D-printed polymer landscape design products. There is, therefore, a need to investigate aspects of 3D-printed polymer landscape design, including laser polishing, material selection, technology aspects, thermodynamic analysis, and surface properties.

In this work, the research objectives are to improve the surface smoothness and mechanical properties of FDM-fabricated PLA landscape design products using laser polishing technology, which are currently under discussion throughout a cooperating interdisciplinary network of materials scientists and engineers. The thermodynamics and rapid solidification during the laser polishing process are analyzed using finite element simulation. Additionally, special attention is paid to the surface morphology of the polished layers, as well as the tensile and flexural properties of the laser-irradiated zone. Finally, we take a 3D-printed PLA Eiffel Tower as a landscape design example to compare the surface morphology before and after laser polishing.

## 2. Materials and Methods

**Figure 2:**
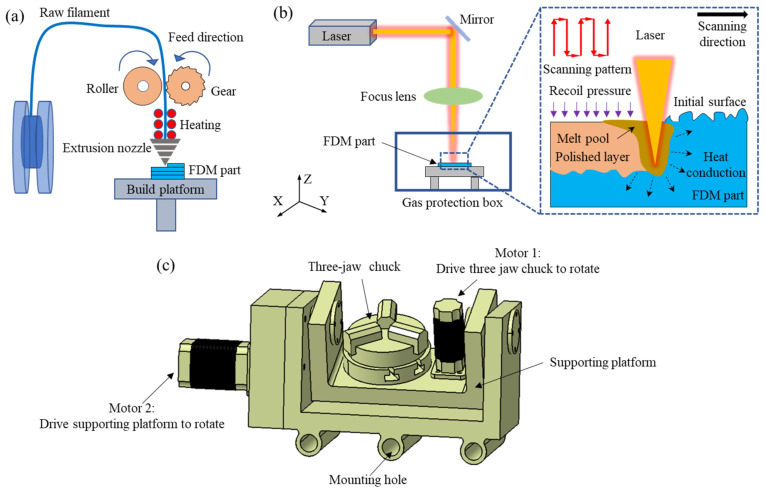
The process for 3D-printed PLA components: (**a**) schematic representation of FDM process; (**b**) schematic representation of laser polishing; (**c**) rotary machining platform.

The FDM equipment (MakerBot^®^ ReplicatorTM 2X, MakerBot Industries, Brooklyn, NY, USA) was used to print the tensile and flexural specimens. The raw material used in this experiment was pure PLA thermoplastic wire (1.75 ± 0.1 mm) from FLASHFORGE Corp. China. The parameters of the FDM used in this paper are listed in Table 1, which referred to [12]. The FDM principle is displayed in Figure 2a, and the raw filament is fed into the extrusion nozzle by the driving force generated from a drive roller and a gear. When the extrusion nozzle moves in the X-Y axis, the raw filament is heated by heating coils to a semiliquid state and is extruded by the extrusion head at the same time. After the completion of the first layer, the platform moves down or the extrusion head moves up one layer along the Z axis. Through this process, the specimen will be deposited layer-by-layer until the part is completed. The detailed dimensions for the tensile and three-point flexural tests were taken from ASTM D638-10 and ASTM D790-10, respectively [12].

Then, according to the reference [20,21,34,35,36], a continuous mode fiber laser with optimized parameters (SPI red ENERGY G4, wavelength 1070 nm, power 5 W, scanning speed 1200 mm/s, overlapping ratio 35%, defocus amount 35 mm and spot size 100 µm) was utilized to polish the specimens. Because the hydroxyl group of PLA can be oxidized by oxygen to form pyruvic acid under the conditions of high temperature heating, the color of pyruvic acid will gradually darken in the air. Therefore, we used the Ar gas to protect the specimens during polishing process. The reaction formula was as follows [37]:

2CH_3_-CHOH-COOH (polylactic acid) + O_2_ = 2CH_3_-CO-COOH (pyruvic acid) + 2H_2_O (1)

The tensile and flexural specimens were clamped by a rotating three-jaw chuck and laser-polished while rotating on all four sides. A schematic diagram of the laser polishing process is shown in Figure 2b. The loading device and its method of movement is shown in Figure 2c, in which motor 1 can drive the three-jaw chuck to rotate, the three-jaw chuck and motor 1 are mounted on the support platform, and motor 2 can drive the support platform to rotate. The laser scanning pattern is a “snake” scanning mode and the scanning pattern is located by the red light mounted on the laser.

A 1/10,000 precision balance (WT-1000) (WANTE Weighing Apparatus Co. Ltd., Hangzhou, China) was used to measure the mass values of the specimens. Tensile and three-point flexural tests were performed using a SANS electronic universal testing machine (Zhongte Machinery Co. Ltd., Jinan, China). The experimental parameters of the tests are listed in Table 2. Five specimens were tested until failure occurred and the results were averaged. A 3D laser scanning confocal microscope (LSCM, VK100, Keyence, Osaka, Japan) was used to measure the surface topography and roughness, with a cutoff wavelength of 650 nm and profile length of 1.5 mm when measuring Ra. The cross-section, surface morphology, and specimen fracture with different magnifications of the polished surface were characterized using an SU8010 scanning electron microscope (SEM), in which all specimens were sprayed with gold. An intelligent infrared thermal imager (YRH300, Nikon, Tokyo, Japan) was used to monitor the laser polishing in real time, for which the measurement resolution, minimum measurement size, temperature range, and sensing distance were 640 × 480 pixels, 20 × 15 cm^2^, 10–2500 °C, and 20–500 mm, respectively.

The temperature during the laser polishing process was measured using the numerical simulation method. The commercial ABAQUS software (ABAQUS 2019, SIMULIA, Detroit, RI, USA) was used to measure the temperature distribution at the surface of the PLA during the laser melting. The physical parameters of the materials were temperature-dependent and the processing parameters were identical to the experiments, for which the thermophysical parameters of the PLA reference were taken the literature [13,38], which can be seen in Table 3. Heat transport through the bottom walls was neglected (adiabatic walls) and convective flow was dominant in energy transmission during the laser polishing process. Because the physic process of laser polishing is very complex in molten pools, some assumptions were formulated to simplify the model: (1) the molten flow was laminar, and the liquid material was considered as a Newtonian and incompressible fluid; (2) the vapor in this model was considered as non-conductive; (3) the Ar gas was neglected in the polishing process; (4) only the melting and solidification process of the molten pool were considered. The polishing model was calculated on a rectangular base measuring 3 × 1 × 0.5 mm^3^. The minimum differential volume unit was a cube measuring 20 × 20 × 20 μm^3^. In the sequential thermal analysis, 56,578 elements were used to create the model. The calculation used linear elements to solve the heat equation. Additionally, the rest of the information about the convergence criteria, boundary conditions, and governing equations were reported by He et al. [39]. The input heat flux density of each spot was calculated using the Gaussian function [40].

(2) $$q=\frac{2P}{\pi r^{2}}\times eff\times exp\left(-2\times \frac{\left[{\left(x_{1}-x_{0}\right)}^{2}+{\left(y_{1}-y_{0}\right)}^{2}\right]}{r^{2}}\right)$$

where *q*, *P*, and *r* are the heat flux density, laser power, and spot radius, respectively; *eff* (46.5%) is the laser absorption rate; *x*_0_ and *y*_0_ are the instantaneous positions of the spot center; $x_{1}$ and $y_{1}$ are the variables of the position.

## 3. Results and Discussion

### 3.1. Simulation Results for the Temperature Distribution

**Figure 3:**
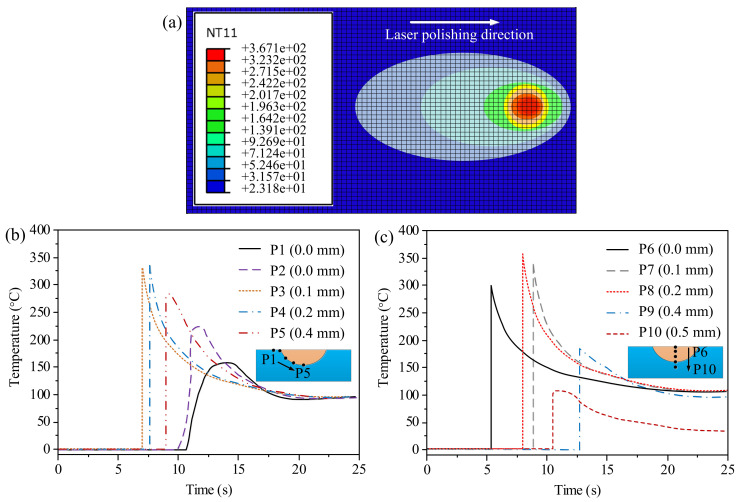
Numerical simulation and thermodynamic analysis of FDM-fabricated PLA using laser polishing: (**a**) Temperature field simulation; (**b**) temperature profiles at different depths on the surface of molten pool; (**c**) temperature profiles at different depths on the center of molten pool.

The temperature distribution of the cross-section of the polished layer is shown in Figure 3a,c. As shown in Figure 3a, the temperature distribution proves that the molten pool was elliptical in shape due to the high-speed movement of the laser source and the maximum temperature was about 367.1 °C at the center of molten pool. The molten pool and the heat-affected zone (HAZ) had temperatures of 201.7 °C and 92.6 °C, respectively. Compared to the melt pool and the HAZ, the top surface of the melted zone had a higher temperature value, which could cause vaporization at the top surface of the polished layer. Further, we measured the temperature curves of five different points at the melt–substrate interface of the polishing layer, as well as the temperature curve changes with time, as shown in Figure 3b. It can be seen that the maximum temperatures were 150.31, 225.33, and 327.56 °C, respectively, at points P1, P2, and P3. At point P4, the maximum temperature was predicted to be 347.58 °C; the boiling point temperature of PLA is less than this value. At point P5, which is the bottom of the melted zone, the maximum was 274.91 °C; no vaporization happened at this point due to the temperature of P5 being lower than the melt pool temperature. Figure 3c reveals the center temperature of the cross-section of the polished layer, indicating that the time for each point to reach the maximum temperature was distinct. It can be seen that the maximum temperatures were 305.39, 349.27, 366.56, 198.21, and 111.99 °C at points P6, P7, P8, P9, and P10, respectively. The maximum temperature increased first and then decreased from the top surface to the bottom of the molten pool, indicating that the highest temperature along the cross-section existed in the middle of the molten pool and not at the top surface. Additionally, each of these points reached its peak temperature at a different time. It can be seen that from the beginning of polishing process, points 9 and 10 were dormant, then they were activated in turn with the evolution of the molten pool. From the simulation temperature distribution, we can conclude that the cooling rates were varied with the change of the melt depth. Additionally, the scanning speed reached 1200 mm/s, which determined the interaction time. In this case, the surface ablation was limited and only vaporization occurred [20]. The polymer appeared not to shrink (no obvious ripple on the polished surface) along the direction of solidification, indicating that the polished specimen had better mechanical consistency than the as-received specimen. We need to point out that in the actual measurement process, we only measurd the surface temperature of the molten pool, while the internal temperature could not be obtained using actual measurements. In addition, we ignored the convective flow, the influence of argon, and the change of density during polishing. 

### 3.2. Laser Polishing of Tensile Specimen

**Figure 4:**
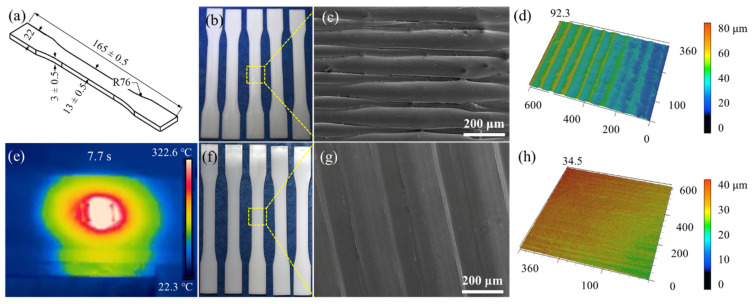
The surface morphology of tensile specimens before and after laser polishing: (**a**) specimen size; (**b**,**c**) macroscopic and microscopic surface morphologies of as-received specimen; (**d**) 3D topographic image of as-received specimen; (**e**) real-time monitoring of temperature field; (**f**,**g**) macroscopic and microscopic surface morphologies of laser-polished specimen; (**h**) 3D topographic image of laser-polished specimen.

Figure 4a,b show the tensile dimensions and a macro-scale photograph, respectively. There are obvious filamentous wavy and seam lines between the layers on the as-received surface (Figure 4c), which led to delamination and high surface roughness. The macro photograph of the laser polishing using the above process parameters is shown in Figure 4f. It is noted that the FDM-fabricated ripples on the as-received surface were significantly reduced via laser polishing, while no obvious defects appeared on the polished surface (Figure 4g). LSCM was used to measure the surface roughness and topography of the polished area, for which the average roughness decreased from over Ra 15 µm to less than 0.25 µm (Figure 4d,h). Additionally, in situ observation is known to play a significant role in investigating laser–matter interactions. Hence, real-time temperature monitoring was adopted to measure the polishing temperature in order to avoid damaging the specimen when the temperature was too high, as the maximum polishing temperature is 322.6 °C at 7.7 s (Figure 4e). Laser surface re-melting is an important physical phenomenon during the laser polishing interactions, which involves re-scanning and re-melting solidified layers, during which an extremely shallow molten pool is produced in a very short time, reducing the viscosity of material [21]. As shown in Figure 2b, the molten phase will be driven away from the convex peaks and flow into the valleys via gravity and surface tension [22]. Meanwhile, recoil pressure occurs on the molten surface due to vaporization of the material when the temperature reaches boiling point, during which the recoil pressure further flattens the melted PLA surface. Eventually, the liquid PLA material will be solidified high cooling rates of approximately 410 K/s and over [41], leading to a significant reduction of the surface roughness.

### 3.3. Laser Polishing of Flexural Specimen

**Figure 5:**
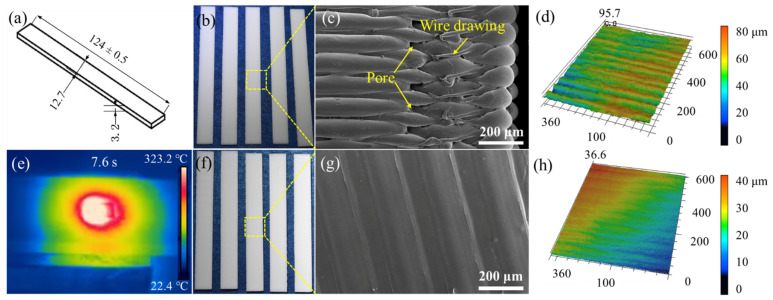
Surface morphology of flexural specimens before and after laser polishing: (**a**) specimen size; (**b**,**c**) macroscopic and microscopic surface morphologies of as-received specimen; (**d**) 3D topographic image of as-received specimen; (**e**) real-time monitoring of temperature field; (**f**,**g**) macroscopic and microscopic surface morphologies of laser-polished specimen; (**h**) 3D topographic image of laser-polished specimen.

As with the tensile specimens shown in Figure 4, the laser polishing of the flexural specimen is shown in Figure 5. Figure 5a,b show the flexural dimensions and macro-scale photograph, respectively. Similar to Figure 4c, there are also obvious filamentous wavy and seam lines between layers on the as-received surface in Figure 5c. Additionally, a series of pores and wire drawings appear at the edge of the as-received surface (Figure 5c), which was due to the different sizes of the tensile and flexural specimens in the thickness direction, resulting in the different G-Code files and deposition paths [42]. In this process, the maximum polishing temperature was 323.2 °C at 7.6 s (Figure 5e), which was not significantly different to the maximum temperature of tensile polishing process shown in Figure 4e. Additionally, the polished surface showed no obvious defects (Figure 5g), whereby laser irradiation melted the staircase in the as-received surface and removed the pores in between during a step known as densification or sintering. The smooth mechanism of this process is outlined in Section 3.1. The average roughness decreased from Ra 18.45 µm to less than 0.25 µm (Figure 4d,h).

Since the FDM model is approximated by a series of triangles (*.STL format), it leads to a chordal approximation error that will produce a tolerance equal to ±0.2 mm overall [43]. In order to determine whether material removal occurs on the specimen after laser polishing, the commonly mass method was used to analyze the mass loss, which is listed in Table 4. It can be concluded that the as-received mass of the tensile specimen was scattered between 8.4319 g and 8.5455 g, while for the flexural specimen the was between 6.3322 g and 6.4899 g, for which the mass deviations were less than 5% with slight mass variation, indicating that the machining capacity of the FDM printer meets the experimental requirements [44]. After polishing, the mass values of the specimens were reduced, with the mass of tensile specimen being scattered between 8.3890 g and 8.4237 g and for flexural specimen between 6.2145 g and 6.4015 g. It can be inferred that the mass reduction in the polished specimen was due to vaporization at high temperature, which we will discuss in Section 3.4. Meanwhile, recoil pressure from the vaporization and recoil effects due to laser heating also promote weight reduction.

To provide more stable and precise results, the standard deviation of the mass datum from arithmetic mean values was calculated using Formula (2) [12]:

(3) $$S=\sqrt{\left[\left|x_{1}-\bar{x}\right|+\left|x_{2}-\bar{x}\right|+\left|x_{3}-\bar{x}\right|+\cdots +\left|x_{n}-\bar{x}\right|\right]÷\left(n-1\right)}$$

where *S* is the standard deviation, *n* is the number of all data, and $\bar{x}$ is the average value of *x*_1_, *x*_2_, *x*_3_, …, *x_n_*. The average value and standard deviation of the tensile and flexural specimens are listed in Table 5. The results show that the polished specimen has less standard deviations relative to the as-received specimen. In the FDM process, there is a pause due to the exchange of raw material in the molten state, whereby the as-received specimen will produce a wire drawing and waviness region during the pause, which will lead to mass deviation in the as-received specimen. Further, the polishing process with melting and solidification will eliminate the wire drawing and waviness effect, reducing the standard deviations.

### 3.4. Mechanical Properties and Fracture Interface

**Figure 6:**
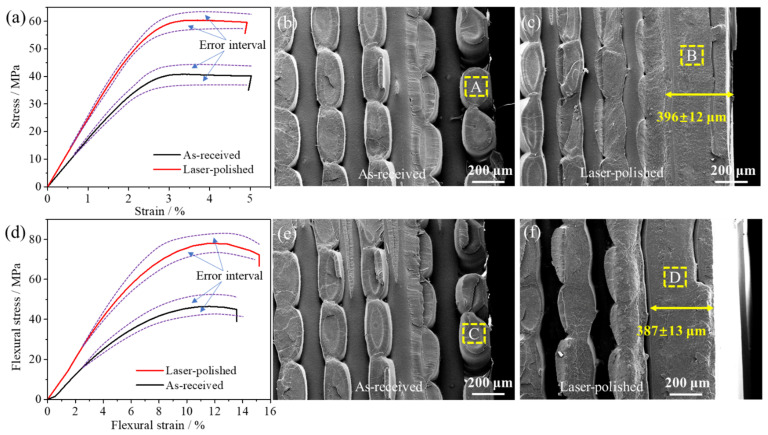
Tensile and flexural tests: (**a**) tensile stress–strain curves; (**b**) tensile SEM fracture of as-received specimen; (**c**) tensile SEM fracture of laser-polished specimen; (**d**) flexural stress–strain curves; (**e**) flexural SEM fracture of as-received Figure 7. Flexural properties and standard deviation; (**f**) flexural SEM fracture of Laser-polished. (A, B, C, D will be enlarged in Figure 7).

Typical tensile stress–strain curves of the as-received and polished samples at room temperature are shown in Figure 6a. There is a clear yield platform in the stress–strain curves, including a linear region and non-linear region. According to the maximum stress value of the non-linear region, the ultimate tensile strength (UTS) can be calculated. Additionally, according to the linear part of the linear fitting, the Young’s modulus can be calculated. The standard deviation of the tensile strength and Young’s modulus is calculated using Formula (2). As shown in Table 6, the polished sample exhibits a higher UTS (average value: 58.57 MPa) than the as-received specimen (average value: 39.96 MPa). Under such processing conditions, the tensile strength can be increased by up to 46.6%, which exceeds the carbon fiber specimen [45]. Additionally, the Young’s modulus of the polished sample is 1.61 GPa, which is increased by up to 37.6% compared with that of the as-received PLA sample (1.17 GPa). Meanwhile, the lower standard deviation also indicates that laser polishing of the 3D-printed PLA can result in more stable mechanical properties. As shown in Figure 6a, the inexistence of necking and plastic deformation at tensile failure can be verified by the later part of the two stress-strain curves, during which the stress did not change with increasing strain. Further, the tensile SEM fracture can be seen in Figure 6b,c, in which the fracture is smooth with no obvious emergence of plastic deformation, showing brittle failure characteristics consistent with the stress–strain curves in Figure 6a. In this process, the crack propagates along the longitudinal tension until the specimen is divided into two parts. Figure 6c shows that the thickness of the polished layer was 396 ± 12 μm; moreover, pores and vacancies disappeared in the polished layer owing to rapid melting and the solidification process. This morphology eliminates the stress concentration and mechanical dispersion caused by pores and discontinuity of the specimen, which is the main reason for the tensile strength improvement of the specimen [13,36].

The typical flexural stress–strain curves of the as-received and polished specimens at room temperature are exhibited in Figure 6d. The flexural properties and standard deviation are listed in Table 7. Similar to the tensile experiment, the polished flexural sample also exhibited a higher ultimate flexural strength (average value: 75.61 MPa) than the as-received sample (average value: 43.32 MPa), which was increased by up to 74.5%. Additionally, the flexural modulus of the polished sample was 17.66 GPa, which was increased by up to 23.2% compared with that of the as-received PLA sample (14.33 GPa). The lower standard deviation also indicated that laser polishing of the 3D-printed PLA can lead to more stable flexural properties. The material melts and solidifies under the influence of laser radiation, which will affect the standard deviation and let the experimental data congregate more. As a surface treatment process, laser polishing can solve the problem of “control performance” for high-performance polymer 3D printing components. The initial stage of the flexural stress–strain curves can be defined as a simple linear relationship, when the specimen is in the process of slight linear elastic deformation. However, once the sample is destroyed, the stress is quickly decreased, showing obvious brittle fracture characteristics. This failure morphology is confirmed in Figure 6e,f. The fracture surface is smooth and without cracks, while the thickness of the flexural polished layer is 387 ± 13 μm. Differing from the as-received flexural specimen, the first crack of the polished specimen occurs at the internal vacancy point when the specimen bends to failure. However, the initial failure of the as-received specimen occurs at the surface due to the existence of pores, which are the weak points of the stress concentration.

**Figure 7:**
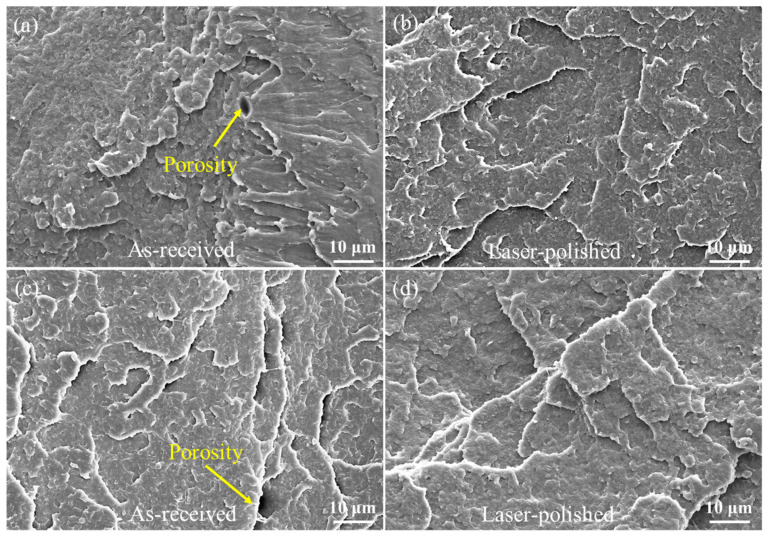
SEM fracture: (**a**) tensile SEM fracture of the as-received specimen; (**b**) tensile SEM fracture of the laser-polished specimen; (**c**) flexural SEM fracture of the as-received specimen; (**d**) flexural SEM fracture of the laser-polished specimen. The panels (**a**–**d**) in this figure correspond to Figure 5a–d, respectively.

In order to characterize the fracture morphology, a clearer, magnified view of the fracture is shown in Figure 7, whereby Figure 7a–d are correspond to Figure 6a–d, respectively. The high magnification fracture surfaces of the laser-polished and as-received specimens are relatively uniform, while the morphological analysis reveals the presence of porosity on the as-received fracture surfaces (Figure 7a,c). The reasons for this phenomenon are that the errors appear at the actual feed rate and the preset rate during the manufacturing process, which leads to the imbalance of interfacial adhesion between the layers. The stable mobility of the molten polymer shifts the PLA material from a rigid state to flexible solidification, thereby influencing the internal microstructure [13]. Additionally, other studies have shown that the cooling process from the molten to the solid state during the FDM process can cause porosity production due to the shrinkage of the specimen [42]. As shown in Figure 7b,d, the polished fracture is similar to the as-received specimen, which is a typical brittle fracture. The reason for the slight change of the fracture after laser treatment is that the laser does not destroy the molecular structure of the PLA and weakens the interaction between molecular chains, thus leading to the similar fracture morphology. The interaction between the laser and the material includes not only evaporation, but also melting [22]; polymer decomposition occurred in both the liquid and softened phases, blowing up from the inner regions and driving the development of bubbles. The bubbles expanded up to overflow on the surface, reducing the above porosity; this effect was expected.

### 3.5. Examples of Laser Polishing on Landscape Design Products

**Figure 8:**
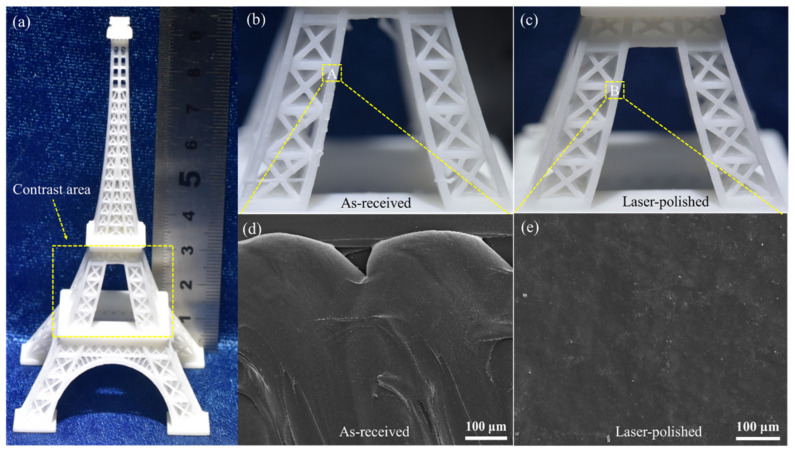
The surface morphology before and after laser polishing of the landscape design example: (**a**) 3D-printed PLA Eiffel Tower; (**b**) as-received specimen; (**c**) laser-polished specimen; The bottom panels (**d**,**e**) correspond to the top panels (**a**,**b**).

Designers’ understanding of “landscape design” is based on the concept or goal of “an ideal sustainable form”. In summary, new urban landscapes clearly will benefit from the methods and knowledge of 3D printing combined with laser polishing in the future. If this interdisciplinary integration can be achieved, cities can develop ecologically to achieve urban sustainability, whereby the model set of real estate shown in Figure 1 will be easier to construct. These strategies aim to link the landscape design experiments with more advanced practical technology. Therefore, a 3D-printed PLA Eiffel Tower is shown in Figure 8a as a landscape design example to compare the surface morphology before and after laser polishing. The model as simulated and optimized using a series of scales. It can be seen from the macro images in Figure 8b,c that the polished surface becomes smoother, without serrated ripples or wire drawing. According to the SEM amplification in Figure 8d,e, the deposition traces on the as-received surface disappear and the polished surface presents a flatter morphology. It can be seen that the applications of scaled 3D printing combined with laser polishing in the building lifecycle of the traditional landscape model is feasible and valuable.

## 4. Conclusions

This work demonstrates the potential of laser polishing to enhance the surface quality and mechanical properties of 3D-printed polymer specimens. The laser polishing process has significantly reduced the shortcomings of 3D printing, such as the high surface roughness and porosity of as-fabricated FDM surfaces; the surface roughness can be reduced from over Ra 15 µm to less than 0.25 µm. Additionally, the polished specimen showed lower standard deviation relative to the as-received specimen, while no shrinkage of cavities or pores or microcracks were found in the laser-polished layer, which had a thickness of 396 ± 12 μm. Both the tensile and flexural strength were increased by up to 46.6% and 74.5%, respectively, compared with as-fabricated components due to the reduction of shortcomings after laser polishing, while the mass reduction in the polished specimen was due to vaporization at high temperature. Moreover, the FEM temperature distribution showed that the peak temperature increased first and then decreased from the top surface to the bottom of the molten pool. Through this kind of interdisciplinary research, hot spots and research needs are emerging, providing sufficient motivation for those researchers who are ready to carry out landscape design studies in the future. Furthermore, our next major research task will be to study the service life of 3D-printed polymer landscape design products involving laser polishing technology, including the fatigue life.

## Figures and Tables

**Table 1:** FDM parameters used in this paper.

Parameter	Value
Object infill density	100%
Layer height	0.1 mm
Feed rate	33 mm/s
Travel feed rate	58 mm/s
Print temperature	210 °C
Filament diameter	1.75 ± 0.1 mm
Nozzle diameter	0.3 mm
Deposition infill pattern	±45°

**Table 2:** Experimental parameters of the tensile and flexural test.

Categories	Tensile Test	Flexural Test
Testing speed	1.5 mm/min	1.5 mm/min
Sampling frequency	20 Hz	20 Hz
Distance between two grips	110 mm	/
Distance between two supports	/	50 mm

**Table 3:** The input parameters during FEM simulation.

Parameter	Value
Laser power	5 W
scanning speed	1200 mm/s
spot size	100 µm
Density	1280 Kg/m^3^
Thermal conductivity	0.025 W/m·K
Melting point	450.15 K
Solid specific heat capacity	1950 J/(Kg·K)
Liqud specific heat capacity	2120 J/(Kg·K)

**Table 4:** Mass analysis of the specimens.

Categories	Tensile Specimens (Unit: g)					Flexural Specimens (Unit: g)				
As-Received	8.4722	8.5262	8.4319	8.5455	8.5113	6.5322	6.4723	6.3842	6.4026	6.5899
Laser-Polished	8.4013	8.4237	8.3997	8.3890	8.3973	6.3545	6.4015	6.3328	6.3803	6.3992

**Table 5:** The average value and standard deviation of the tensile and flexural specimens.

Categories	Tensile Specimens (Unit: g)		Flexural Specimens (Unit: g)	
	$\bar{x}$	*S*	$\bar{x}$	*S*
As-received	8.4974	0.2130	6.4762	0.2912
Laser-polished	8.4022	0.1037	6.3737	0.1732

**Table 6:** Tensile properties and standard deviation.

Categories	Young’s Modulus (GPa)	Young’s Modulus Standard Deviation (GPa)	Tensile Strength (MPa)	Tensile Strength Standard Deviation (MPa)
As-received	1.17	0.26	39.96	0.73
Laser-polished	1.61	0.05	58.57	0.52

**Table 7:** Flexural properties and standard deviation.

Categories	Flexural Modulus (GPa)	Flexural Modulus Standard Deviation (GPa)	Flexural Strength (MPa)	Flexural Strength Standard Deviation (MPa)
As-received	17.66	0.46	43.32	0.65
Laser-polished	14.33	0.23	75.61	0.37

## Data Availability

The data presented in this study are available on request from the corresponding author.
